# Supplementary material for: Splice-Junction-Based Mapping of Alternative Isoforms in the Human Proteome
Source: Cell Rep. Author manuscript; Available in PMC 2020 Jan 15. (PMC6961840; doi:10.1016/j.celrep.2019.11.026)
Supplement: 2 [file NIHMS1546469-supplement-2.zip › DF1/PXD010154/Heart/Heart_44_TTN_KVEAPPPKVPEVPK.pdf]

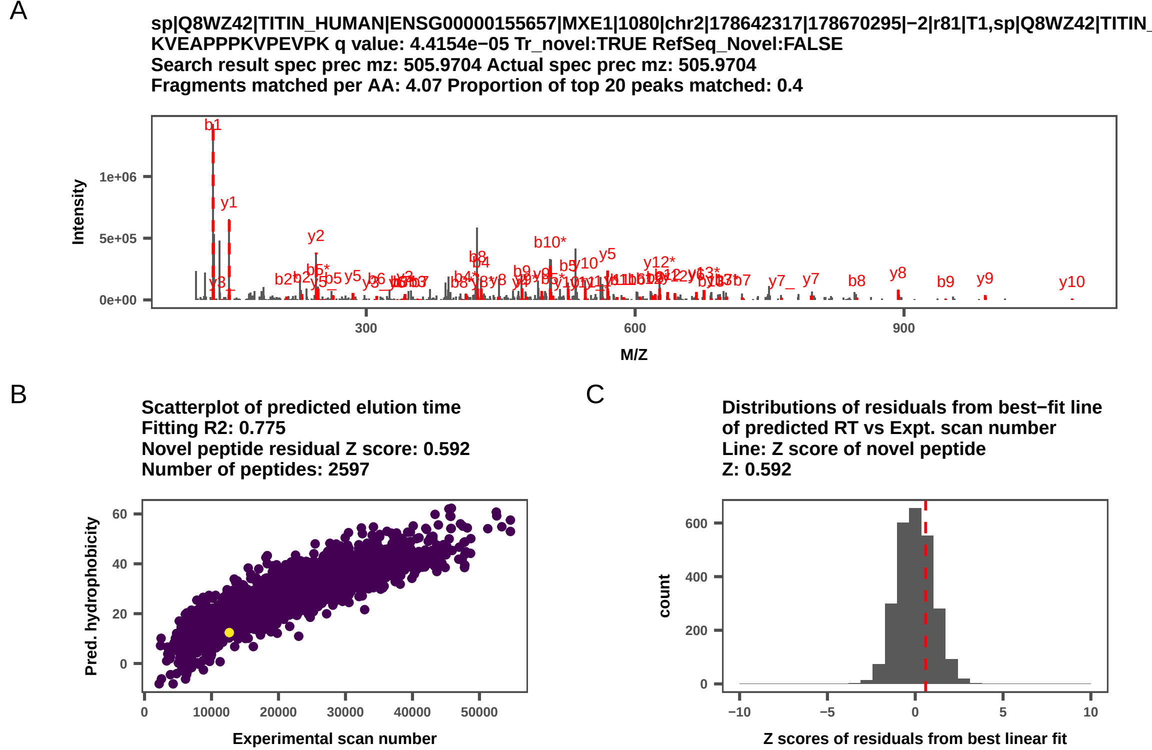
A
sp|Q8WZ42|TITIN_HUMAN|ENSG00000155657|MXE1|1080|chr2|178642317|178670295|−2|r81|T1,sp|Q8WZ42|TITIN_
KVEAPPPKVPEVPK q value: 4.4154e−05 Tr_novel:TRUE RefSeq_Novel:FALSE
Search result spec prec mz: 505.9704 Actual spec prec mz: 505.9704
Fragments matched per AA: 4.07 Proportion of top 20 peaks matched: 0.4
Intensity
1e+06
5e+05
0e+00
b1
y1
y2
b10*
y5
y7_
y7
b8
y8
b9
y9
y10
300
600
900
M/Z
B
Scatterplot of predicted elution time
Fitting R2: 0.775
Novel peptide residual Z score: 0.592
Number of peptides: 2597
Pred. hydrophobicity
60
40
20
0
0
10000
20000
30000
40000
50000
Experimental scan number
C
Distributions of residuals from best−fit line
of predicted RT vs Expt. scan number
Line: Z score of novel peptide
Z: 0.592
count
600
400
200
0
−10
−5
0
5
10
Z scores of residuals from best linear fit
